# Supplementary material for: Structural basis of epilepsy-related ligand–receptor complex LGI1–ADAM22
Source: Nat Commun. 2018 Apr 18;9:1546. doi: 10.1038/s41467-018-03947-w (PMC5906670; doi:10.1038/s41467-018-03947-w)
Supplement: Supplementary file 1 — Supplementary Information [file 41467_2018_3947_MOESM1_ESM.pdf]

Supplementary Information

Structural basis of epilepsy-related ligand–receptor complex LGI1–ADAM22

Yamagata *et al.*

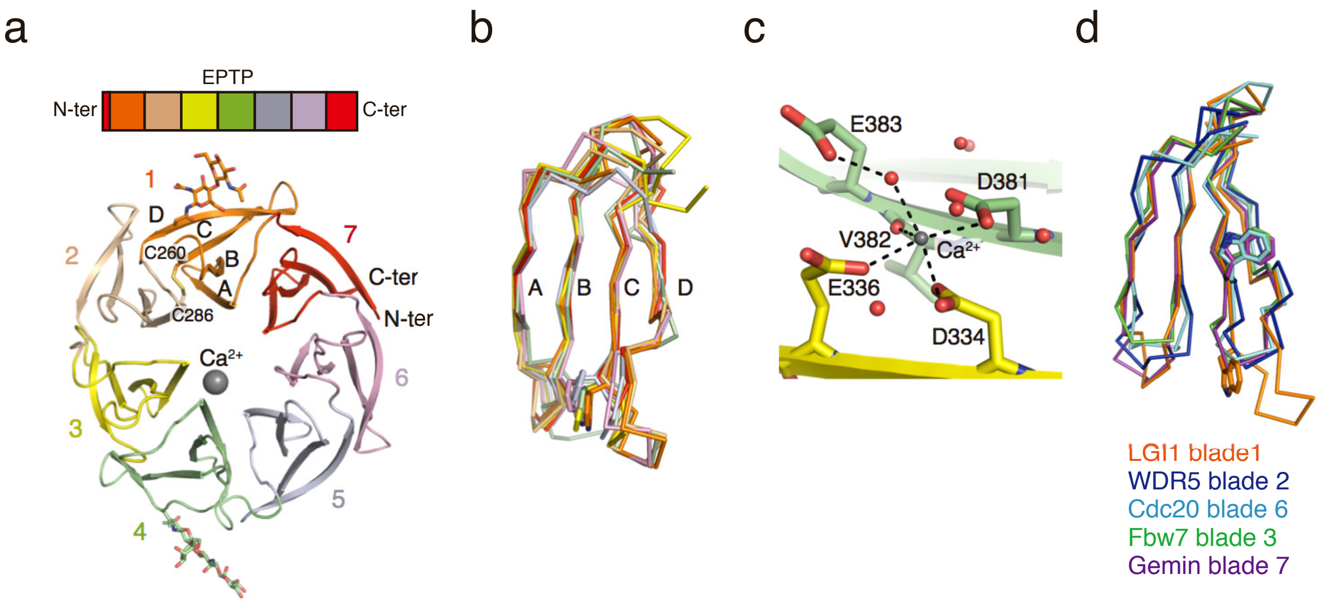

e

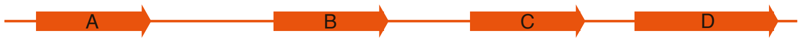


```
                                                       WD
blade1    235     QSLSIDTFSYL------NDEYVVIAQPFT-GKCIFLEWDHVEKTFRNYDNITG   280
blade2    281    TSTVVCKPIVIE------TQLYVIVAQLFG--GSHIYKRDSFANKFIKIQDIEI   326
blade3    327 LKIRKPNDIETFKIE------NNWYFVVADSSKAGFTTIYKWNG--NGFYSHQSLHA   375
blade4    376   WYRDTDVEYLEIVRTPQTLRTPHLILSSSSQ--RPVIYQWNKATQLFTNQTDIPN   428
blade5    429   MEDVYAVKHFSVK------GDVYICLTRFIG--DSKVMKWGG--SSFQDIQRMPS   473
blade6    474    RGSMVFQPLQIN------NYQYAILGSDYS--FTQVYNWDAEKAKFVKFQELNV   519
blade7    520    QAPRSFTHVSIN------KRNFLFASSFKG--NTQIYKHV   TEFAKSQDLPY   234
consensus              hp h hp       pp ahhh           hapa      a  hp h
```

Supplementary Figure 1 β-propeller structure of LGI1 EPTP.

(a) Seven-bladed β-propeller architecture of LGI1 EPTP. Four strands in blade 1 are labeled as A–D. The Cys260–Cys286 disulfide bond is shown as sticks. The bound Ca^{2+} is shown as a grey sphere.

(b) Overlay of seven blades of the LGI1 EPTP β-propeller. Five tryptophan residues in WD-like motifs are shown as sticks.

(c) Ca^{2+}-binding site in the LGI1 EPTP β-propeller. Ca^{2+} and water molecules are shown as grey and red spheres, respectively.

(d) Overlay of blades in the LGI1 EPTP β-propeller and other typical WD40 proteins. The blades of WDR5 (PDB 5SXM), Cdc20 (PDB 4GGA), Fbw7 (PDB 2OVR), and Gemin (PDB 5TEE) are aligned. Tryptophan residues in WD motifs are shown as sticks.

(e) Structure-based sequence alignment of seven blades in the LGI1 EPTP β-propeller. Consensus sequences are shown in the bottom of the alignment (h, hydrophobic; p, polar; a, aromatic). The position of WD-like motifs is indicated above the alignment. Strands A–D are represented in the top.

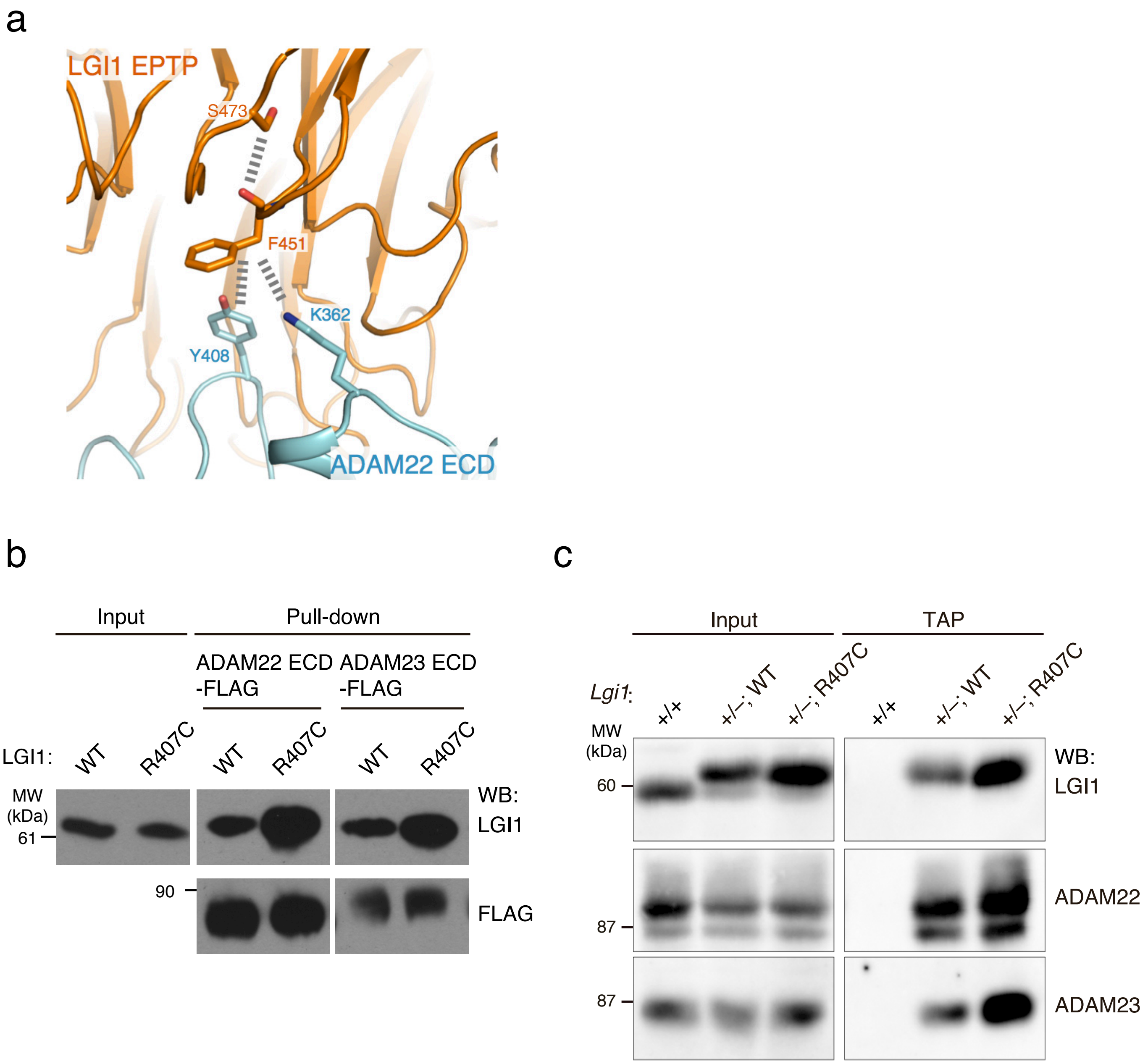

Supplementary Figure 2 Secretion-competent LGI1 mutants, LGI1^{S473L} and LGI1^{R407C}.

(a) Close-up view of the area around Ser473 of LGI1 in the interface between LGI1 EPTP and ADAM22 ECD. Dotted lines represent putative steric clashes occurring in LGI1^{S473L} and between LGI1^{S473L} and ADAM22 ECD.

(b) Pull-down assay between LGI1^{WT} or LGI1^{R407C} and ADAM22 or ADAM23 ECD-FLAG. LGI1^{WT} or LGI1^{R407C} and ADAM ECD-FLAG secreted from HEK293T cells were mixed and pulled down with FLAG antibody agarose. Shown are Western blots of input (left) and (co-)purified (right two panels) samples with indicated antibodies.

(c) Tandem-affinity purification (TAP) of LGI1^{WT} and LGI1^{R407C} tagged with FLAG and His_6 from the mouse brain, showing that ADAM22 and ADAM23 were enriched with purified LGI1^{WT} and LGI1^{R407C} similarly. Shown are Western blots of input (left) and TAP eluates (right) with indicated antibodies.

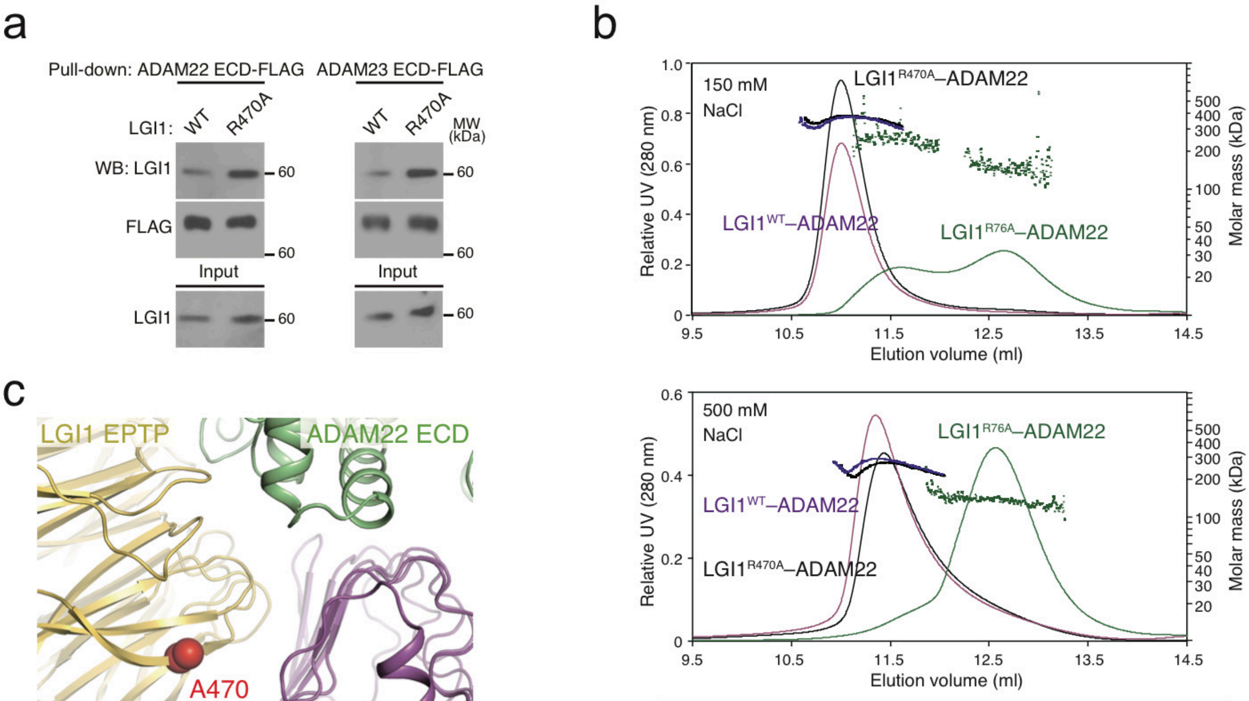

LGI1–ADAM22	Molar mass (kDa) [NaCl] (mM) 150	500	Theoretical Molar mass (kDa)
LGI1^{WT}–ADAM22	356	267	234 (2:2 complex)
LGI1^{R470A}–ADAM22	367	258	234 (2:2 complex)
LGI1^{R76A}–ADAM22	252 157	137	117 (1:1 complex)

Supplementary Figure 3 Characterization of LGI1^{R470A}.

(a) Pull-down assay between LGI1^{WT} or LGI1^{R470A} and ADAM22 or ADAM23 ECD-FLAG. LGI1^{WT} or LGI1^{R470A} and ADAM ECD-FLAG secreted from HEK293T cells were mixed and pulled down with FLAG antibody agarose. Shown are Western blots of the (co-)purified (upper two panels) and input (bottom) samples with indicated antibodies.

(b) SEC-MALS analyses of LGI1^{WT}–ADAM22 ECD (purple), LGI1^{R470A}–ADAM22 ECD (black) and LGI1^{R76A}–ADAM22 ECD (green) at 150 mM or 500 mM NaCl. Chromatograms and determined molar masses are shown.

(c) Close-up view of the area around Ala470 of LGI1^{R470A} in the 2:2 LGI1^{R470A}–ADAM22 complex.

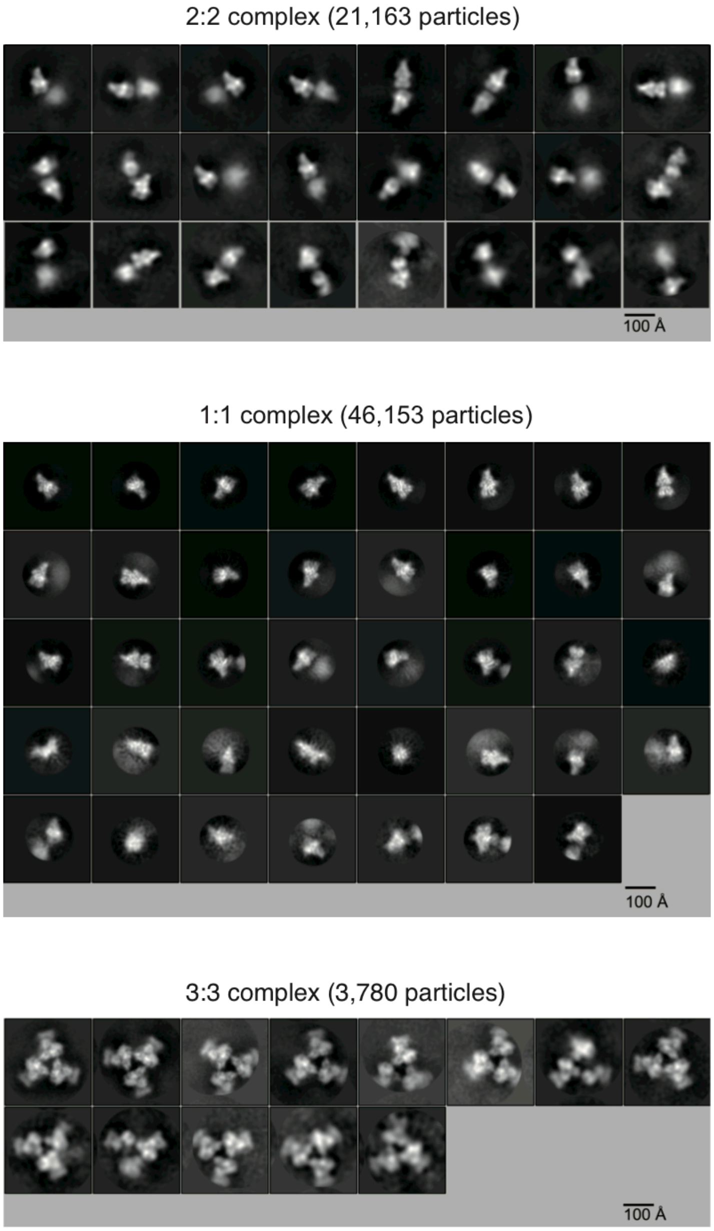

Supplementary Figure 4 Cryo-EM of LGI1^{R470A}–ADAM22 complex.

Reference-free 2D class averages corresponding to the 2:2, 1:1 and 3:3 LGI1^{R470A}–ADAM22 complexes are shown.

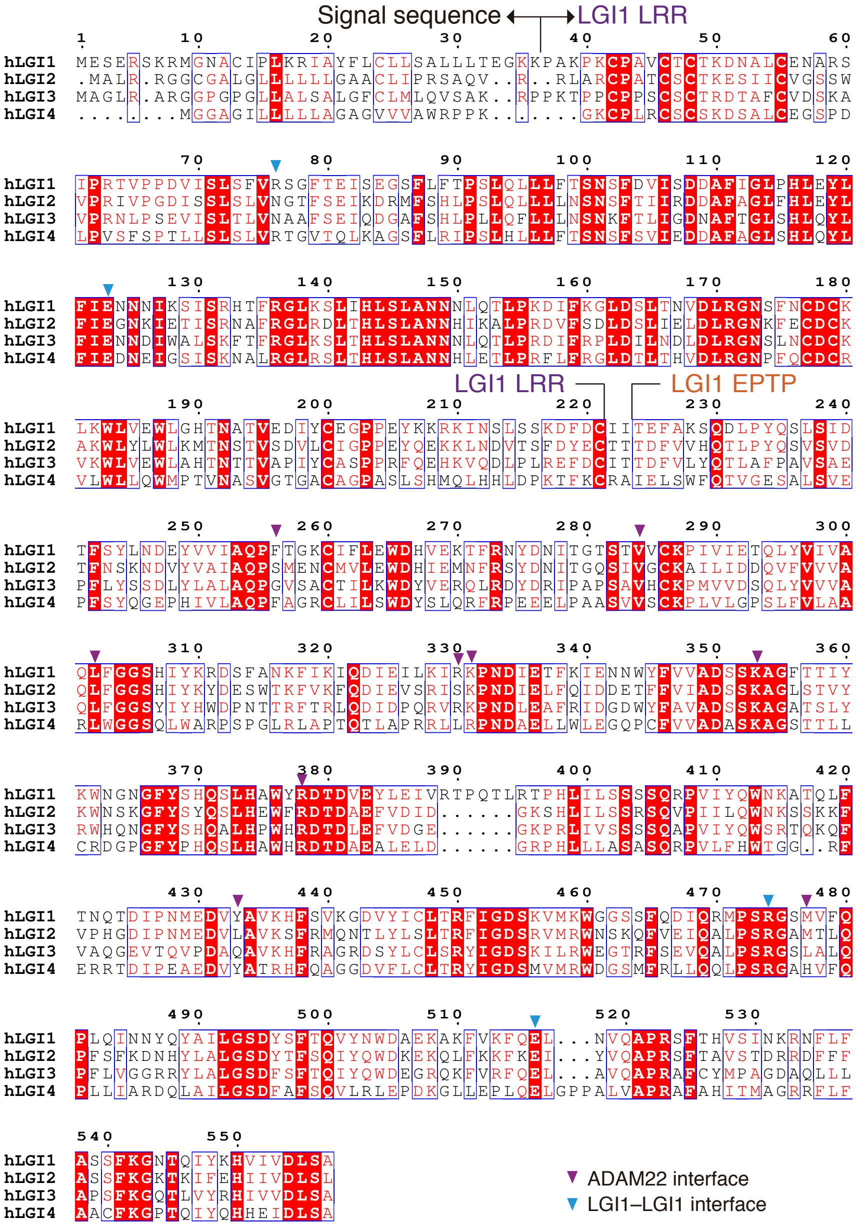

Supplementary Figure 5 Amino-acid sequence alignment of human LGI1–LGI4.

The residues involved in the LGI1 EPTP–ADAM22 and LGI1–LGI1 interfaces in the 2:2 LGI1–ADAM22 complex are indicated by inverted magenta and cyan triangles, respectively.

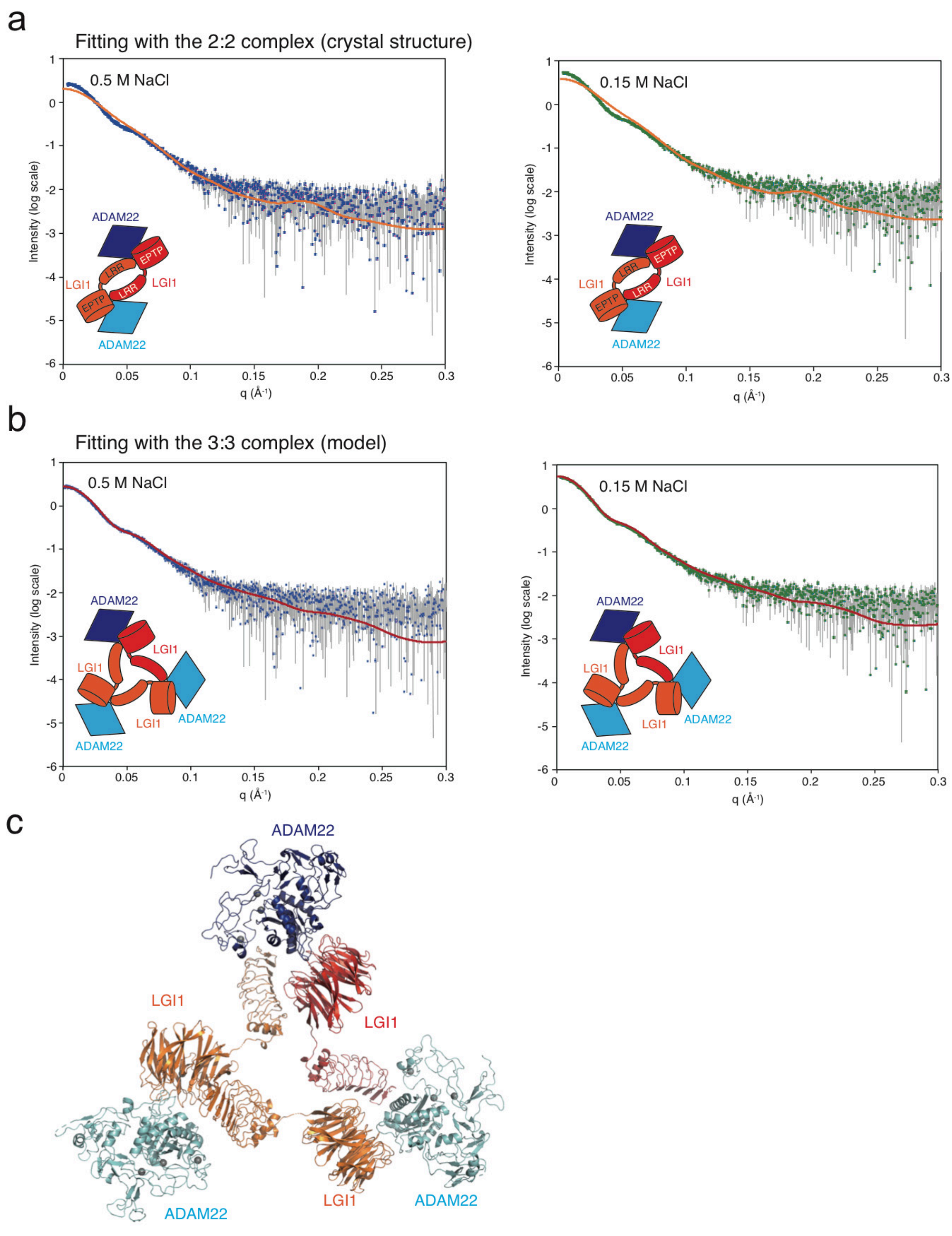

Supplementary Figure 6 SEC-SAXS analysis of LGI1–ADAM22 complex.

(a,b) Measured scattering curves of the LGI1–ADAM22 complex at 500 mM and 150 mM NaCl. The theoretical scattering curve calculated from the crystal structure of the 2:2 LGI1–ADAM22 complex **(a)** or that from the model structure of the 3:3 LGI1–ADAM22 complex **(b)** is overlaid.

(c) Modeled structure of the 3:3 LGI1–ADAM22 complex with the pseudo *C*3 symmetry.

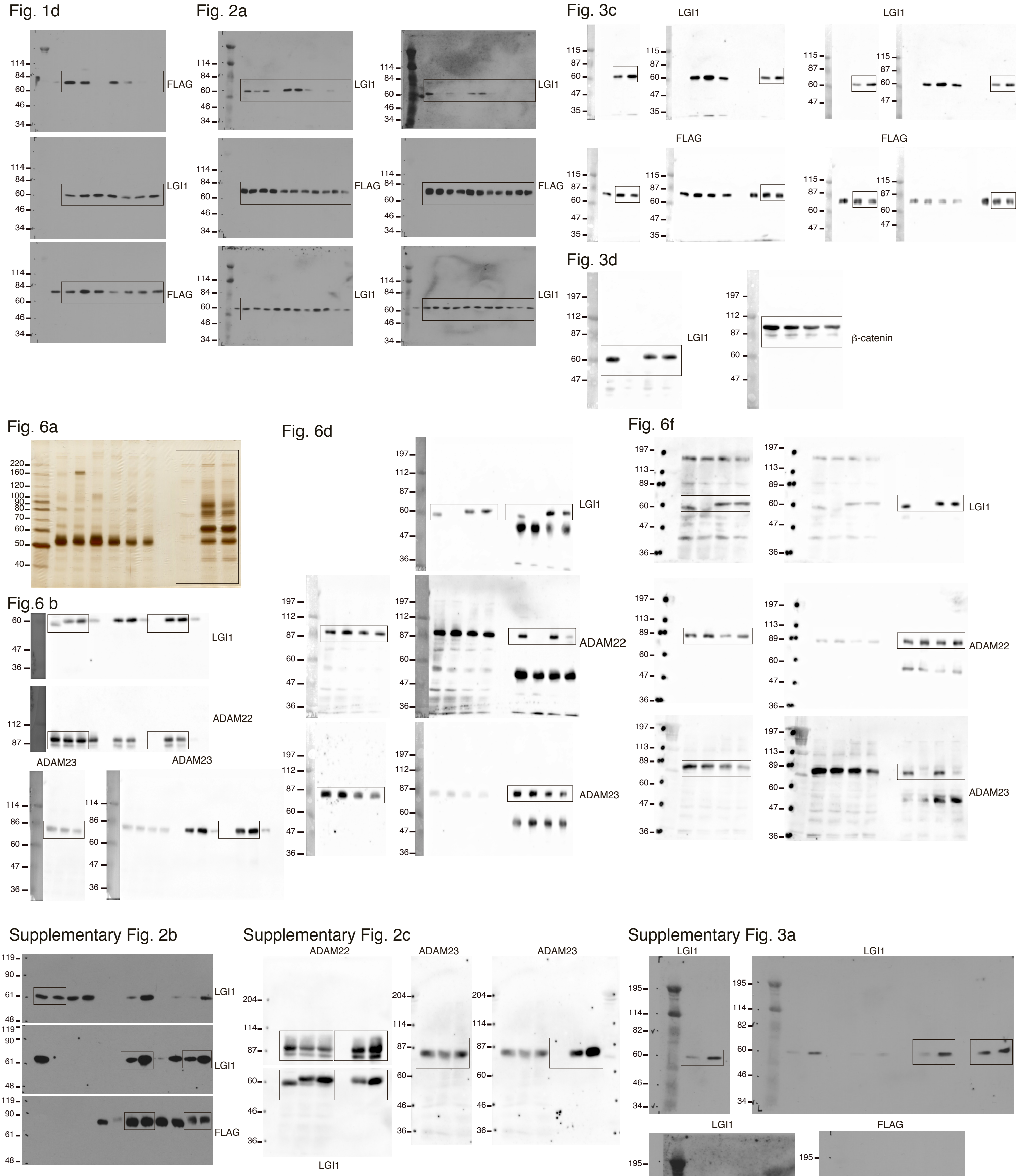

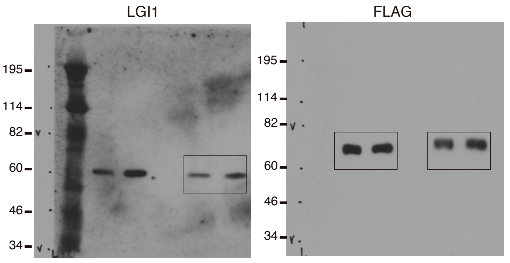

Supplementary Figure 7 Uncropped images of blots and gels.

Supplementary Table 1 Summary of 28 ADLTE missense mutations on the LGI1 structure.

Mutations	Nucleotide	Domain	Position	Structural/functional effects	Secretion	Reference
C42R	124T>C	LRR-NT	N-terminal Cys cap	Disruption of intramolecular disulfide bond with C48	Defective	1-3
C42G	124T>G	LRR-NT	N-terminal Cys cap	Disruption of intramolecular disulfide bond with C48	Defective	2-4
C46R	136T>C	LRR-NT	N-terminal Cys cap	Disruption of intramolecular disulfide bond with C55	Defective	2,3,5,6
C46F	137G>T	LRR-NT	N-terminal Cys cap	Disruption of intramolecular disulfide bond with C55	Not examined	7
I82T	245T>C	LRR 1	Structural core	Misfolding	Not examined	8
A110D	329C>A	LRR 2	Structural core	Misfolding	Defective	1-3
I122K	365T>A	LRR 3	Structural core	Misfolding	Defective	2,3,9
I122T	365T>C	LRR 3	Structural core	Misfolding	Defective	2,10
E123K	367G>A	LRR 3	Inter-LGI1 interface	Misfolding	Defective	2,3,11
R136W	406C>T	LRR 3	Structural core	Misfolding	Defective	2,3,10
S145R	435C>G	LRR 4	Structural core	Misfolding	Defective	2,3,12
L154P	461T>C	LRR 4	Structural core	Misfolding	Defective	2,3,13
C179R	535T>C	LRR-CT	C-terminal Cys cap	Disruption of intramolecular disulfide bond with C221	Defective	2,10
C200R	598T>C	LRR-CT	C-terminal Cys cap	Disruption of intramolecular disulfide bond with C177	Defective	2,3,14
L214P	641T>C	LRR-CT	Structural core	Misfolding	Not examined	15
F226C	677T>G	EPTP-Blade 7	Structural core (β-strand D)	Misfolding	Not examined	16
L232P	695T>C	EPTP-Blade 7	Structural core (β-strand D)	Misfolding	Defective	2,3,17
C286R	856T>C	EPTP-Blade 2	Structural core (β-strand A)	Disruption of intramolecular disulfide bond with C260	Not examined	18
I298T	893T>C	EPTP-Blade 2	Structural core (β-strand B)	Misfolding	Defective	1-3
F318C	953T>G	EPTP-Blade 2	Structural core (β-strand D)	Misfolding	Defective	2,3,19
L373S	1118T>C	EPTP-Blade 3	Structural core (β-strand D)	Misfolding	Not examined	18
T380A	No data	EPTP-Blade 4	Structural core (β-strand A)	Misfolding	Defective	2,20
E383A	1148A>C	EPTP-Blade 4	Structural core (β-strand A /Ca^{2+} coordination)	Misfolding	Defective	2,3,21
R407C	1219C>T	EPTP-Blade 4	Surface (Loop B-C)	unaffected	Competent	2,22
V432E	1295T>A	EPTP-Blade 5	Structural core (Loop 4D-5A)	Misfolding	Defective	2,3,14
S473L	1418C>T	EPTP-Blade 5	Structural core (Loop 5D-6A)	Disruption of the binding to ADAM22	Competent	2-4,23
R474Q	1421G>A	EPTP-Blade 6	Inter-LGI1 interface (Loop 5D-6A)	Disruption of LGI1 dimer formation	Competent	2,23
G493R	1477G>A	EPTP-Blade 6	Structural core (Loop B-C)	Misfolding	Defective	2,24

Supplementary Table 2 SEC-SAXS data.

	LGI1–ADAM22 ECD	
[NaCl]	150 mM	500 mM
**Data collection parameters**		
Instrument	BL45XU	BL45XU
Wavelength (Å)	1.0000	1.0000
q range ($Å^{-1}$)[a]	0.0033-0.3027	0.0033-0.3027
Exposure time (sec)	0.25 × 4	0.25 × 4
Concentration (g L^{-1})	0.90	0.67
**Structural parameters**		
I(0) (cm^{-1}) [from Guinier]	5.48±0.013	2.72±0.01
R_g (Å) [from Guinier]	69.43±0.50	67.82±3.91
I(0) (cm^{-1}) [from P(r)]	5.48±0.0108	2.73±0.0095
R_g (Å) [from P(r)]	69.80±0.14	68.79±0.23
D_{max} (Å)	210	209
Porod volume estimate ($Å^3$)	713,000	646,000
M.W. calculated with Porod volume[b]	445,625	403,750
**Software employed**		
Primary data reduction	DataProcess	DataProcess
Data processing	PRIMUS	PRIMUS
Rigid body modeling	SASREF	SASREF
Computation of model intensities	CRYSOL	CRYSOL
Three-dimensional graphics representation	PyMOL	PyMOL

[a] The *q* range used for solution structural determination
[b] M.W. =0.625×Porod volume

Supplementary References

1 Ottman, R. *et al.* LGI1 mutations in autosomal dominant partial epilepsy with auditory features. *Neurology* **62**, 1120-1126 (2004).

2 Yokoi, N. *et al.* Chemical corrector treatment ameliorates increased seizure susceptibility in a mouse model of familial epilepsy. *Nat. Med.* **21**, 19-26 (2015).

3 Nobile, C. *et al.* LGI1 mutations in autosomal dominant and sporadic lateral temporal epilepsy. *Hum. Mutat.* **30**, 530-536 (2009).

4 Berkovic, S. F. *et al.* LGI1 mutations in temporal lobe epilepsies. *Neurology* **62**, 1115-1119 (2004).

5 Gu, W., Brodtkorb, E. & Steinlein, O. K. LGI1 is mutated in familial temporal lobe epilepsy characterized by aphasic seizures. *Ann. Neurol.* **52**, 364-367 (2002).

6 Pizzuti, A. *et al.* Epilepsy with auditory features: a LGI1 gene mutation suggests a loss-of-function mechanism. *Ann. Neurol.* **53**, 396-399 (2003).

7 Lee, M. K. *et al.* A newly discovered LGI1 mutation in Korean family with autosomal dominant lateral temporal lobe epilepsy. *Seizure* **23**, 69-73 (2014).

8 Sadleir, L. G. *et al.* Seizure semiology in autosomal dominant epilepsy with auditory features, due to novel LGI1 mutations. *Epilepsy Res.* **107**, 311-317 (2013).

9 Striano, P. *et al.* A novel loss-of-function LGI1 mutation linked to autosomal dominant lateral temporal epilepsy. *Arch. Neurol.* **65**, 939-942 (2008).

10 Di Bonaventura, C. *et al.* Low penetrance and effect on protein secretion of LGI1 mutations causing autosomal dominant lateral temporal epilepsy. *Epilepsia* **52**, 1258-1264 (2011).

11 Di Bonaventura, C. *et al.* Drug resistant ADLTE and recurrent partial status epilepticus with dysphasic features in a family with a novel LGI1mutation: electroclinical, genetic, and EEG/fMRI findings. *Epilepsia* **50**, 2481-2486 (2009).

12 Hedera, P. *et al.* Autosomal dominant lateral temporal epilepsy: two families with novel mutations in the LGI1 gene. *Epilepsia* **45**, 218-222 (2004).

13 Pisano, T. *et al.* Abnormal phonologic processing in familial lateral temporal lobe epilepsy due to a new LGI1 mutation. *Epilepsia* **46**, 118-123 (2005).

14 Michelucci, R. *et al.* Autosomal dominant lateral temporal epilepsy: clinical spectrum, new epitempin mutations, and genetic heterogeneity in seven European families. *Epilepsia* **44**, 1289-1297 (2003).

15 Klein, K. M. *et al.* Autosomal dominant epilepsy with auditory features: a new LGI1 family including a phenocopy with cortical dysplasia. *J. Neurol.* **263**, 11-16 (2016).

16 Fumoto, N. *et al.* Novel LGI1 mutation in a Japanese autosomal dominant lateral temporal lobe epilepsy family. *Neurol Clin Neurosci* **5**, 44-45 (2017).

17 Chabrol, E. *et al.* Two novel epilepsy-linked mutations leading to a loss of function of LGI1. *Arch. Neurol.* **64**, 217-222 (2007).

18 Dazzo, E. *et al.* Autosomal dominant lateral temporal epilepsy (ADLTE): novel structural and single-nucleotide LGI1 mutations in families with predominant visual auras. *Epilepsy Res.* **110**, 132-138 (2015).

19 Fertig, E., Lincoln, A., Martinuzzi, A., Mattson, R. H. & Hisama, F. M. Novel LGI1 mutation in a family with autosomal dominant partial epilepsy with auditory features. *Neurology* **60**, 1687-1690 (2003).

20 Leonardi, E. *et al.* A computational model of the LGI1 protein suggests a common binding site for ADAM proteins. *PLoS One* **6**, e18142 (2011).

21 Kalachikov, S. *et al.* Mutations in LGI1 cause autosomal-dominant partial epilepsy with auditory features. *Nat. Genet.* **30**, 335-341 (2002).

22 Striano, P. *et al.* Familial temporal lobe epilepsy with psychic auras associated with a novel LGI1 mutation. *Neurology* **76**, 1173-1176 (2011).

23 Kawamata, J. *et al.* Mutations in LGI1 gene in Japanese families with autosomal dominant lateral temporal lobe epilepsy: the first report from Asian families. *Epilepsia* **51**, 690-693 (2010).

24 Heiman, G. A. *et al.* Evaluation of depression risk in LGI1 mutation carriers. *Epilepsia* **51**, 1685-1690 (2010).
