# Supplementary material for: Structural basis of epilepsy-related ligand–receptor complex LGI1–ADAM22
Source: Nat Commun. 2018 Apr 18;9:1546. doi: 10.1038/s41467-018-03947-w (PMC5906670; doi:10.1038/s41467-018-03947-w)
Supplement: Supplementary file 3 — Description of Additional Supplementary Files [file 41467_2018_3947_MOESM3_ESM.pdf]

Description of Additional Supplementary Files

File Name: Supplementary Data 1
Description: Primer sequences used in this study
